# Scattering engineering in continuously shaped metasurface: An approach for electromagnetic illusion

**DOI:** 10.1038/srep30154

**Published:** 2016-07-21

**Authors:** Yinghui Guo, Lianshan Yan, Wei Pan, Liyang Shao

**Affiliations:** 1 Center for Information Photonics & Communications, School of Information Science & Technology, Southwest Jiaotong University, Chengdu, Sichuan, 610031, China

## Abstract

The control of electromagnetic waves scattering is critical in wireless communications and stealth technology. Discrete metasurfaces not only increase the design and fabrication complex but also cause difficulties in obtaining simultaneous electric and optical functionality. On the other hand, discontinuous phase profiles fostered by discrete systems inevitably introduce phase noises to the scattering fields. Here we propose the principle of a scattering-harness mechanism by utilizing continuous gradient phase stemming from the spin-orbit interaction via sinusoidal metallic strips. Furthermore, by adjusting the amplitude and period of the sinusoidal metallic strip, the scattering characteristics of the underneath object can be greatly changed and thus result in electromagnetic illusion. The proposal is validated by full-wave simulations and experiment characterization in microwave band. Our approach featured by continuous phase profile, polarization independent performance and facile implementation may find widespread applications in electromagnetic wave manipulation.

There are considerable interests in controlling and engineering the scattering electromagnetic fields from metallic objects especially for military applications^1^,^2^,^3^. The scattering property of an object is quantitatively determined by virtue of its radar cross section (RCS), which decides how far the object can be detected by a radar system. Traditionally RCS reduction methods rely on modifying the object geometry^4^ and/or loading the object with absorbing materials. Recently, the rapid development of transformation optics^5^,^6^, a powerful means to steer the light flow in almost arbitrary ways, provides a unique design paradigm for electromagnetic cloaks, which bend light around a concealed region, rendering any object inside the region “invisible”. Besides, when combined with plasmonics, transformational optics can be used to realize sub-diffraction limit imaging and optical lithography^7^,^8^,^9^,^10^, which is a tremendous advancement of optics in this century^11^. Unfortunately, the general materials required by transformational optics are in general highly inhomogeneous and anisotropic metamaterials, which significantly constrains the realization and application of practical devices and results in disadvantages of narrow operation bandwidths and high losses^12^,^13^. A quasi-conformal mapping method was subsequently developed^14^,^15^, where only a modest range of isotropic refractive indices is required. Subsequently, electromagnetic cloak based on the near-zero refractive index metamaterials was also investigated^16^. Nevertheless, metamaterial-based schemes are bulky and hard to scale up^17^.

The gradient phase metasurface^18^,^19^,^20^, because of the reduced dimension and loss as well as powerful scattering engineering ability, is taken as a promising approach for electromagnetic cloak or electromagnetic illusion^4^,^17^,^21^, a strategy used to make an object of arbitrary shape and material properties appear exactly like another object of some other shape and material makeup. In 2005, Luo *et al*. proposed the first gradient flat metasurface lens based on surface plasmon^11^. Since its ability to revise the classic Snell’s law and form image beyond the diffraction limit, such lens is expected to be the next-generation optical devices^11^,^22^ . In 2011, Capasso’s group demonstrated another flat lens based on V-shaped nano-antenna array, which could also be considered as a type of metasurface^18^. In general, the behavior of light in gradient metasurface can be described by the metasurface-assisted law of refraction and reflection (MLRR)^11^. Another important gradient metasurface is based on the polarization conversion in anisotropic metasurfaces and the spin-orbit interaction (SOI) in inhomogeneous structures^11^. Luo’s group proved that the traditional Fresnel’s equations can be revised via the metasurface/metamirror approach and gave pioneering work on the broadband polarization conversion^11^,^23^,^24^,^25^. Subsequently, these artificial atoms are arranged in homogeneously to obtain nearly perfect broadband virtual shaping^4^. Theoretically, both the gradient metasurface and the surface absorber can be understood in the scenario of energy exchange between the propagating and bounding waves or the so-called “two-wave exchange”^11^,^26^.

Most previous metasurfaces are constructed by a serial of discrete meta-atom/molecules. Gradient phase was approximated by discrete levels of abrupt phase shift. On one hand, it is a significant challenge obtaining high-performance metasurfaces that can operate at visible frequency range with high uniformity. On the other hand, the discontinuous nature inevitably degrades the overall performance of metasurfaces due to the induced phase noise to the scattering fields, for example, decreasing the purity of generated orbit angular momentum and causing aberrations in imaging and focusing system^27^,^28^,^29^. Besides, discrete system is not electrically conductive, thus it is difficult to obtain simultaneous electric and optical functionality. Quite recently, quasi-continuous super-meta-atoms including catenary and trapezoid shaped structure are adopted in metasurfaces to overcome the shortages above^30^,^31^,^32^,^33^,^34^. Especially, the optical catenary^30^, inspired from natural phenomena, is the first realization of continuous linear phase shift covering (0, 2π) in metasurface, which invoke tremendous interests in searching the mystical interlinks between mechanical structures, religion symbols and optical phonmenona^34^,^35^.

Inspired by the electromagnetic virtual shaping and catenary optics^4^,^30^, here we present a metasurface constructed by true continuous super-meta-atoms, so called continuously shaped metasurface. The phase modulation is origin from the SOI in spatially inhomogeneous sinusoidal metallic strips, which redirects the reflected wave with a period-dependent angle with respect to the specular reflection direction. The polarization independent scattering engineering capability of our structure is able to change-reduce or enhance-the monostatic RCS of the underneath object dramatically.

## Results

### Principle of continuous gradient phase

**Figure 1:**
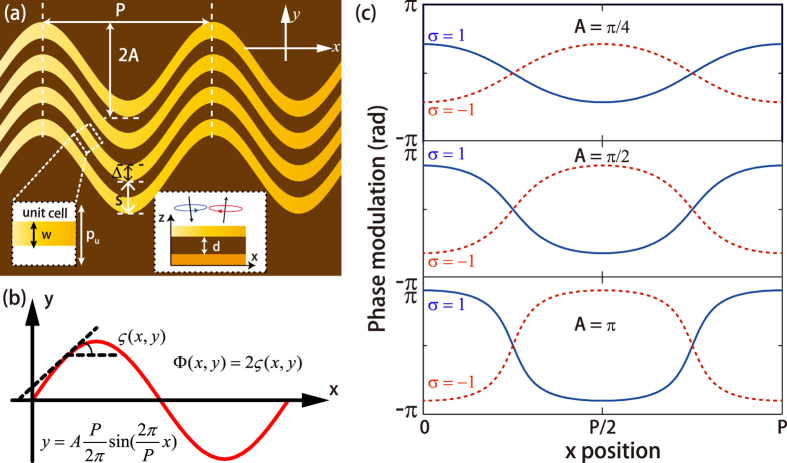
Principle of continuous gradient phase modulatoin based on spin-orbit interaction in space-variant half-wave plates. (**a**) The proposed continuously shaped metasurface is constructed by sinusoidal metallic strip array, which can be considered as a serial of metallic patch with space-variant orientation angle, as shown in the inset. Space-variant half-wave plates are composed of the metasurface, dielectric spacer and a metallic ground plane, which contributes the spin-orbit interaction. (**b**) The phase shift for a rotated half waveplate under CPW. (**c**) Pancharatnam-Berry phase modulation in single period of sinusoidal metallic strip with different amplitude coefficient A.

It is well known that sinusoidal function is the basic oscillating form of electromagnetic wave and the Fourier expansion of an ideal triangular and rectangular waveform can be represented by the summation of sinusoidal functions. Therefore, sinusoids are chosen to realize the continuous phase shift. The geometry of the metasurface is illustrated in Fig. 1(a), where only four rows and two periods of sinusoidal metallic strips are given. As shown in Fig. 1(b), the profile of spatially inhomogeneous metallic strips is mathematically defined by a typical sinusoidal function:


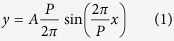


where, *A* and *P* denote the amplitude coefficient and period of the sine curve, respectively.

In our design, a metallic ground plane and a dielectric substrate are added below the metasurface, forming a metamirror as depicted in the inset of Fig. 1(a)^23^,^25^,^36^. When the thickness of the dielectric spacer is adjusted properly, the metasurface, dielectric spacer, and the metallic ground plane would form a space-variant waveplate^4^, thus subjected to the famous SOI. For half-wave plate, circular polarization would be reversed after reflection^36^,^37^. Spatial control of the polarization state of a beam in this manner ultimately introduces spatially varying phase distributions, known to be a manifestation of the Pancharatnam-Berry (P-B) phase^38^, which is just twice the inclination angle between the curve tangent and the *x* axis (Fig. 1(b))^4^


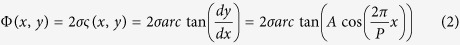


where, σ = ±1 denote the left-handed circular polarization (LCP) and right-handed circular polarization (RCP), respectively. According to the equation (2), the theoretical phase modulation in single period of sinusoidal metallic strip is investigated and illustrated in Fig. 1(c). As we expected, the phase profile is continuous, which may motivate the metasurface-assisted law of reflection/refraction^11^,^22^. Furthermore, the range of phase shift is determined by the amplitude coefficient (*A*) and approaches to the whole range of (−π, π) with the increase of *A*.

### Scattering engineering via continuously shaped metasurface

**Figure 2:**
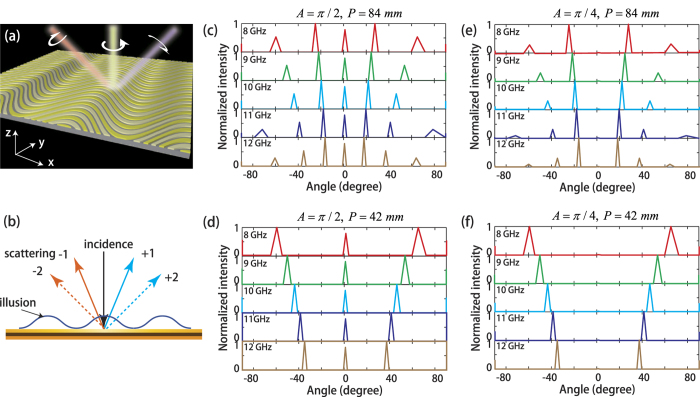
Sketch map of electromagnetic illusion induced by scattering engineering of continuously shaped metasurface. (**a**) Scattering of a planer metamirror under illumination of a CPW. (**b**) Electromagnetic illusion induced by anomalous scattering. (**c**–**f**) Theoretically calculated scattering angular spectra for different designed parameters. (**c**) A = π/2, P = 84 mm. (**d**) A = π/2, P = 42 mm. (**e**) A = π/4, P = 84 mm. (**f**) A = π/4, P = 42 mm.

In order to get physical insight of scattering engineering, we subsequently consider a planer metamirror under normal illumination of circularly polarized waves (CPW) (Fig. 2(a)). Conventional mirrors, known since the dawn of civilization, obey the simple law of reflection. While, the metamirror proposed here due to the gradient phase stemming from the SOI of continuously shaped planar metasurface would force the reflected beam of opposite handedness to propagate in well-defined ways. In this way, traditional law of reflection is broken and rectified by generalized Snell’s law^11^,^18^,^39^, giving rise to electromagnetic illusion as elucidated in Fig. 2(b).

By exploiting the fast Fourier transform (FFT) command in MATLAB^TM^ for the theoretical phase shift in equation (2), the scattering properties of proposed metamirrors with an outer dimension of 20 × 420 mm^2^ are calculated under different circumstances: A = π/2, P = 84 mm (Fig. 2(c)); A = π/2, P = 42 mm (Fig. 2(d)); A = π/4, P = 84 mm (Fig. 2(e)); A = π/4, P = 42 mm (Fig. 2(f)). Evidently, due to the period of the sinusoidal metallic strip is larger than the wavelength of 8–12 GHz, multiple scattering orders emerge and the scattering angular spectra manifest several remarkable features. Initially, for each metamirror, the scattering patterns through 8–12 GHz are similar because of the frequency independent P-B phase, which may pave the avenue for broadband illusion devices. Secondly, the intensity of the scattering orders can be varied by adjusting the amplitude coefficient (*A*) of sinusoidal metallic strips. Especially, 0 order scattering, the main source of RCS, is totally suppressed when A is around π/4, which is incompetent for traditional grating. Ultimately, the deflection angle of m^-th^ scattering order with respect to the specular direction is frequency and period dependent, approximately satisfying:


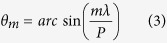


Consequently, various kinds of scattering angular spectra can be obtained with properly adoption of A and P. Note that, although the P-B phase modulation in Fig. 1(c) is polarization sensitive, the scattering characteristics of the proposed metasurface is same regardless of the handedness of CPW and no scattering circular dichroism^40^ is observed here.

### Simulation and experimental validation of electromagnetic illusion

**Figure 3:**
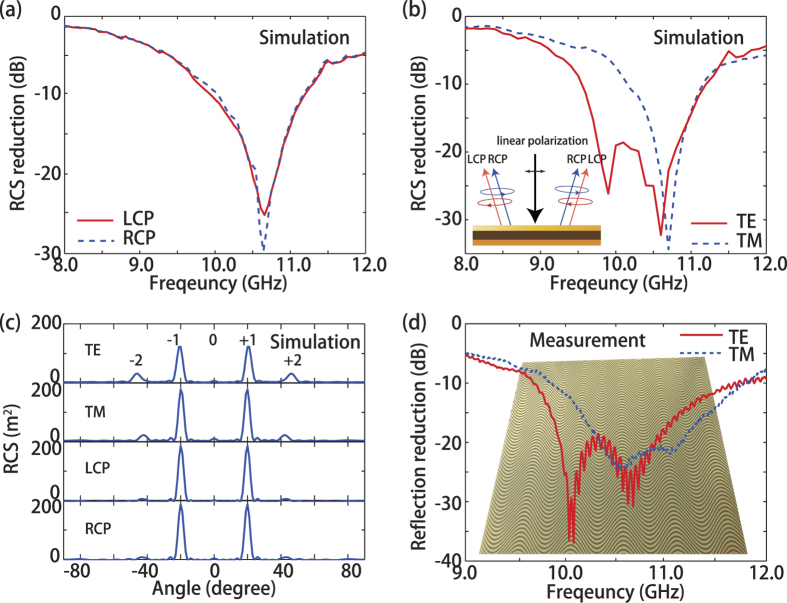
Simulation and experimental characterization of electromagnetic illusion in RCS reduction. (**a**) Simulated RCS reduction of planer metamirror under illumination of LCP and RCP. (**b**) Simulated RCS reduction of planer metamirror under illumination of TE and TM. (**c**) Simulated RCS at 10.6 GHz for different polarizations as a function of angle. (**d**) Measured reflection reduction of planer metamirror under illumination of TE and TM.

The performance of electromagnetic illusion based on proposed metasurface is characterized by the RCS metamorphosis. Full model simulation is carried out for a metamirror with a thickness of 2 mm. The amplitude coefficient (*A*) and period (*P*) of the metallic winding strips is π/4 and 84 mm, respectively. The period of the sinusoidal metallic strips along y direction is s = 6.8 mm, less than the incident wavelength λ to prevent the diffraction in yoz plane. The width of sinusoidal metallic strips is Δ = 4 to obtain high polarization conversion efficiency around 10 GHz. As shown in Fig. 3(a), the monostatic RCS in 10–11 GHz is reduced larger than 10 dB compared with a planar metallic plate for both LCP and RCP. The maximum of the RCS reduction exceeds 25 dB around 10.6 GHz. Combination with the fact that the frequency dependent conversion efficiency of CPW in practice, it is not strange that the operation bandwidth is not broad.

A linear polarized light with an angle of polarization φ can be written as a superposition of its circular components as follows:


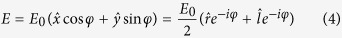


where 
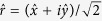
 and 
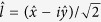
 are the unit vectors of the RCP and LCP, respectively. Similar RCS reduction performances are achieved for the transverse electric (TE) or transverse magnetic (TM) polarization, as illustrated in Fig. 3(b). In addition, the RCS of the metamirror as a function of angle at 10.6 GHz are investigated for different polarizations. The scattering angular spectra presented in Fig. 3(c) also indicate the scattering engineering scheme is polarization independent, which is in contrast to the intuitive thought about anisotropic material. We can see the 0 order scatting is greatly suppressed and ±1 order scattering is mainly directed to ±20°, which is consistent with the theoretical result revealed in Fig. 2(e). In fact, scattering engineering can also be realized by other metasurfaces, for example, continuous triangle grating and discrete metasurfaces. However, the proposed structure in this paper has its advantages. Firstly, this continuously shaped metasurface can be scaled to visible band due to the released fabrication requirement compared with those discrete systems. Besides, the phase accuracy of continuously shaped metasurface is significantly improved owing to the nearly infinite small “pixel” size. In contrast, the “pixel” size of phase modulation is limited by the dimension of super-atoms in discrete metasurfaces. Therefore, it is a great challenge obtaining high-performance discrete metasurfaces that can operate at visible frequency range with high uniformity. Finally, although the triangle grating is continuous, the generated phase profile is discontinuous and broken at the turning point.

In order to prove the numerical results, a sample was fabricated with print circuit board (PCB) technique as shown in the inset of Fig. 3(b). Reflection measurements were taken in a microwave anechoic chamber with a network analyzer. By changing the orientation of linearly polarized horn antennas, the reflectance was measured for both the TE and TM polarizations. The results shown in Fig. 3(d) display 20 dB reflection reduction around 10.6 GHz, which are in reasonable agreement with the simulated results in Fig. (b), considering the imperfection in the fabricating and measuring process.

**Figure 4:**
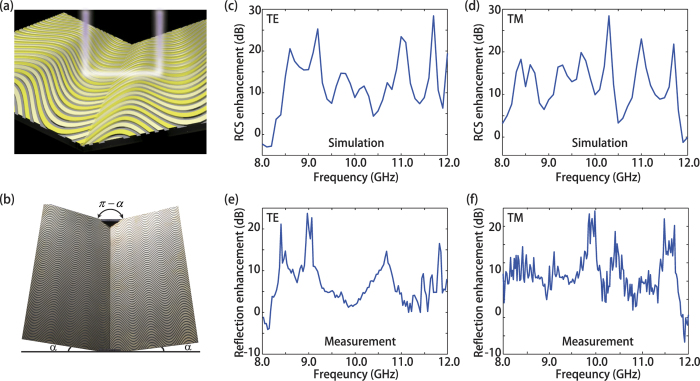
Simulation and experimental characterization of electromagnetic illusion in RCS enhancement. (**a**) A prototype of electromagnetic illusion device for RCS enhancement. (**b**) Photograph of the fabricated samples. (**c**,**d**) Simulated RCS enhancement of dihedral corner reflector under illumination of TE and TM. (**e**,**f**) Measured RCS enhancement under illumination of TE and TM.

Finally, we would like to extend the concept of continuously shaped metasurface to RCS enhancement, dubbed as superscatterers in some literatures^41^,^42^. For example, in wireless communications where reliable communications likely rely on of direction and orientation of the mobile devices, RCS enhancement will enable tracking it with improved signal-to-noise ratio^43^. The configuration of RCS enhancement is presented in Fig. 4(a), where the continuously shaped metasurface is warped over in a symmetrical dihedral corner reflector with a π − 2α opening angle (Fig. 4(b)). Here, α is chosen as π/8 so that the flatness is improved compared to a conventional orthogonal dihedral corner reflector (with a π/2-opening angle, i.e. α = π/4). The monostatic return from such structures can be enhanced when its virtual shape behaves like an orthogonal dihedral corner reflector. The performance of RCS enhancement by proposed continuously shaped metasurface is investigated by full model simulation. The geometric parameters are same with the previous design. As shown in Fig. 4(c,d), the average monostatic RCS enhancement in 8–12 GHz is more than 10 dB for both TE and TM polarizations compared with the cases without metasurface. Note that the RCS enhancement can be further improved by optimizing of period of the sinusoidal metallic strips to make the ±1 order scattering of metasurface is redirected back. A sample was fabricated by PCB technology and fixed on a home-made clamp to engage the opening angle is 3π/4, as shown in Fig. 4(b). The measurement results under orthogonal linear polarizations (TE and TM) are respectively displayed in Fig. 4(e,f), which is consistent with the simulation results. The discrepancy between them arises mainly from the imperfect phase realization and nonuniform scattering amplitudes across the dihedral corner reflector.

## Discussions

In summary, a new generation scheme of continuous gradient phase has been proposed and investigated, which is origin from the spin-orbit interaction in spatially inhomogeneous sinusoidal metallic stripes. Owing to the continuous phase modulation, phase noises in the case of antenna arrays can be avoided. Furthermore, by adjusting the geometric parameters of sinusoidal metallic stripes, we can engineer the gradient phase and make the scattering characteristics of the underneath object deviate from its actual status. Polarization independent electromagnetic illusion devices based on proposed continuously shaped metasurface also have been numerically and experimentally demonstrated in microwave band for significant reduction or enhancement of the scattering cross section of an object, which can be subsequently pushed to the infrared and even the optical range by downscaling the sizes of the structural dimension. Although the geometric phase shift is frequency independent, the operation bandwidth of illusion devices is limited due to the uniform polarization conversion efficiency. It is anticipated that by resorting to the versatile dispersion management strategy and multi-resonance technique presented by Luo’s group^26^,^36^,^44^,^45^,^46^, the operation band will be further expanded. On the other case, the pronounced Fano resonance in metasurface can also be utilized to achieve ultra-narrow band operation^47^. Finally, by utilizing dynamic control method^48^,^49^, the performance of our device can be significantly improved.

## Methods

### Numerical analyses and simulations

MATLAB^TM^ is used as the basic numerical tool for the projects. The scattering patterns of the metamirror were calculated by using the fast Fourier transform command for the theoretical phase distributions in equation (2). The full model simulations were carried out by using commercial software with open boundary conditions. The amplitude coefficient (*A*) and period (*P*) of the metallic winding strips is π/4 and 84 mm, respectively. The period of the sinusoidal metallic strips along y direction is s = 6.8 mm. The width of sinusoidal metallic strips is Δ = 4. In the simulations, perfect electrical conductor (PEC) model was selected for the metallic patterns and the ground plane. The substrate under metallic patterns has a permittivity of 4.5 and thickness of 2 mm.

### Sample fabrication and characterization

The sample with outer dimension (420 mm × 420 mm) was fabricated by using laser direct writing in print circuit board (PCB) technology. The performance of electromagnetic illusion was characterized by the reflection variation between the proposed metamirror and metallic reflection plane with the same configuration. The measurements were carried in a microwave anechoic chamber. Two standard linearly polarized horn antennas as transmitter and receiver, respectively, were connected to the two ports of a vector network analyzer R&S ZVA40. The incident angle was set as 5°, which is a good approximation of the normal incidence. By changing the orientation of linearly polarized horn antennas, the reflectance was measured for both the TE and TM polarizations.

## Additional Information

**How to cite this article**: Guo, Y. *et al*. Scattering engineering in continuously shaped metasurface: An approach for electromagnetic illusion. *Sci. Rep.* **6**, 30154; doi: 10.1038/srep30154 (2016).
